# Synergistic Effects of Glial Fibrillary Acidic Protein Mutation and Overexpression in the Pathogenesis of Alexander Disease

**DOI:** 10.3390/ijms27104405

**Published:** 2026-05-15

**Authors:** Ni-Hsuan Lin, Ming-Der Perng

**Affiliations:** 1 Institute of Molecular Medicine, College of Life Sciences and Medicine, National Tsing Hua University, Hsinchu 30043, Taiwan; nancynihsuan@gapp.nthu.edu.tw; 2 School of Medicine, College of Life Sciences and Medicine, National Tsing Hua University, Hsinchu 30043, Taiwan

**Keywords:** GFAP mutation, Alexander disease, astrocyte dysfunction, proteostatic stress, Rosenthal fibers

## Abstract

Alexander disease (AxD) is a rare and fatal neurodegenerative disorder caused by dominant mutations in the *gfap* gene, which encodes glial fibrillary acidic protein (GFAP), a major intermediate filament in astrocytes. As a primary astrogliopathy, AxD is marked by white matter abnormalities, the formation of GFAP-containing Rosenthal fibers, astrocyte dysfunction, and progressive neurodegeneration. While GFAP mutations are known to cause toxic gain-of-function effects, the precise mechanisms by which mutant GFAP drives astrocyte dysfunction and central nervous system pathology remain unclear. To address this, we developed a novel rat model of AxD harboring the R237H mutation in the endogenous *gfap* locus, which mirrors the R239H mutation commonly associated with early-onset AxD in humans. This model recapitulates key AxD pathologies, including GFAP aggregation, widespread astrogliosis, white matter abnormalities, and motor deficits. Using homozygous mutant rats, we dissected the distinct contributions of mutant GFAP and elevated GFAP expression to astrocyte dysfunction and neurodegeneration. Our findings reveal that AxD pathogenesis results from a synergistic interaction between the toxic gain-of-function properties of mutant GFAP and its elevated expression, which together drive GFAP aggregation, proteostatic stress, and astrocyte dysfunction. These insights provide a deeper understanding of AxD mechanisms and a foundation for developing targeted therapies for this devastating disease.

## 1. Introduction

Alexander disease (AxD, OMIM #203450) is a rare and devastating neurodegenerative disorder caused by dominant mutations in the *gfap* gene, which encodes glial fibrillary acidic protein (GFAP), the primary intermediate filament (IF) protein in mature astrocytes. As one of the few primary disorders of astrocytes, AxD is characterized by progressive white matter abnormalities and a wide spectrum of clinical symptoms, including seizures, developmental delays, cognitive decline, speech and swallowing difficulties, and autonomic dysfunction [1]. The disease can manifest at any age and is typically progressive and fatal. Based on the location of lesions in the central nervous system (CNS), AxD is classified into two clinical subtypes [2]: type 1, which is associated with early-onset disease, with symptoms such as forebrain-predominant lesions, seizures, macrocephaly, and developmental delays; and type 2, which occurs at any age and is characterized by hindbrain-predominant lesions and symptoms such as ataxia, dysphagia, and autonomic dysfunction. Despite these classifications, significant variability in clinical presentation exists, and the mechanisms underlying this heterogeneity remain poorly understood.

Mutations in the *gfap* gene are identified in over 90% of AxD patients, with more than 100 disease-causing variants identified across the 432 amino acids of GFAP’s major isoform, GFAPα [3]. Recent studies have further refined the classification of GFAP variants, highlighting the distribution of mutations across the protein sequence and identifying specific residues as mutation hotspots [4]. Notably, residues such as R79, R88, R239, and R416 are frequently mutated, with the R239H mutation being particularly associated with early-onset AxD [5]. Although most AxD mutations are thought to exert toxic gain-of-function effects, rare cases of recessive inheritance have been reported [6,7,8]. Given that the same mutations had previously been shown to exhibit dominant inheritance in several patients, these cases likely represent dominant inheritance influenced by gene dosage effects. Despite the wide mutational spectrum of GFAP, genotype–phenotype correlations remain poorly defined, complicating efforts to predict disease severity or progression.

The histopathological hallmark of AxD is the presence of Rosenthal fibers, which are ubiquitinated protein aggregates found within astrocytes [9]. These aggregates, essential for AxD diagnosis, are composed primarily of GFAP and other IF proteins, such as vimentin [10], nestin [11], and synemin [12], along with small stress proteins like αB-crystallin and heat shock protein 27 [13], and other components, such as cyclin D2 [14]. Although Rosenthal fibers are observed in other neurological conditions, they are particularly abundant in AxD [15]. While the precise mechanisms underlying their formation remain incompletely understood, it is hypothesized that Rosenthal fibers form when GFAP levels exceed a critical threshold [11,16]. This threshold is believed to be lower in the presence of mutant GFAP [17], which has altered assembly properties and a higher propensity to aggregate [18]. However, whether Rosenthal fibers themselves are the primary toxic species or merely byproducts of a broader pathological process remains unclear. Emerging evidence suggests that the pathological changes associated with Rosenthal fibers may directly impair astrocyte function [19], leading to a cascade of secondary effects on other CNS cell types [20,21]. This positions AxD as a unique model of primary astrogliopathy for elucidating the role of astrocyte dysfunction in neurodegenerative processes [22]. Despite these advances, the precise mechanisms by which mutant GFAP leads to astrocyte dysfunction remain poorly understood. In particular, the interplay between the toxic gain-of-function properties of mutant GFAP and the secondary increase in total GFAP levels caused by reactive gliosis has not been fully elucidated.

To address these knowledge gaps, we developed a novel rat model harboring the R237H mutation in the endogenous *gfap* gene [23]. This mutation is orthologous to the R239H mutation, which is frequently associated with early-onset AxD in humans [5]. This model exhibits hallmark features of AxD, including widespread astrocyte pathology, white matter abnormalities, and motor impairments, making it a powerful tool for dissecting the molecular and cellular underpinnings underlying AxD pathogenesis. In this study, we explore whether the pathogenesis of AxD is driven by a synergistic interaction between the toxic gain-of-function properties of mutant GFAP and the increased GFAP levels due to gene dosage. We hypothesize that mutant GFAP initiates the disease process by disrupting filament assembly, promoting aggregation, and inducing proteostatic stress. This, in turn, triggers gliotic responses that further elevate GFAP expression. This resulting increase in total GFAP levels exacerbates the disease by amplifying proteostatic stress, accelerating aggregate formation, and impairing astrocyte function. This creates a self-perpetuating cycle of astrocyte dysfunction and neurodegeneration. To test this hypothesis, we generated homozygous and heterozygous mutant rat lines and used them to investigate how GFAP mutation and overexpression interact to drive AxD pathogenesis. By comparing these models, we aim to delineate the relative contributions of mutant GFAP and increased GFAP levels to astrocyte dysfunction and disease progression.

## 2. Results

### 2.1. Gene Dosage Effect and Disease Severity

Human AxD is characterized by the presence of GFAP mutations combined with elevated total GFAP levels. However, a key unresolved question is whether astrocyte dysfunction in AxD results from the expression of mutant GFAP alone or from the combined effect of GFAP mutation and increased GFAP levels. Previous studies using AxD model rats with a heterozygous missense mutation replicated some key aspects of the disease but showed a relatively mild phenotype, with approximately 10% mortality by 12 weeks [23]. These findings led us to hypothesize that significant morbidity in AxD might occur when the expression of mutant GFAP exceeds a critical threshold.

**Figure 1:**
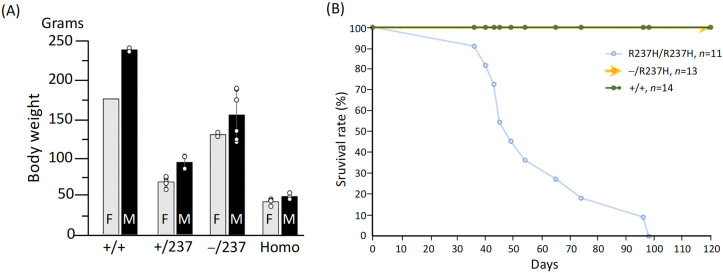
Body weight measurement and survival analysis of rats with R237H GFAP mutation. (**A**) Comparison of body weights among rats of the indicated genotypes. At 7 weeks of age, Homo rats exhibited a significant reduction in body weight compared to their wild-type (+/+) littermates, regardless of sex. Additionally, both Het mutant-null (−/R237H) and Het mutant (+/R237H) rats also showed decreased body weights relative to wild-type (+/+) rats. The number of rats (*n*) ranged from 1 to 5 for each genotype. (**B**) When Het mutant-null (R237H/−) rats were crossed either with each other or with Het mutant (R237H/+) rats, all progeny with the homozygous R237H mutation died by a median age of 58 days. In contrast, WT (+/+) and Het mutant-null (R237H/−) rats exhibited 100% survival for at least 120 days. The numbers in parentheses represent the number of rats evaluated for each genotype.

To test this hypothesis, we generated a cohort of rats by crossing heterozygous (Het) mutant-null (−/R237H) rats either with each other or with Het mutant (+/R237H) rats. Among the progeny, both Het mutant-null (−/R237H) and Het mutant (+/R237H) rats were found to be smaller than WT littermates at 7 weeks of age (Figure 1A). At the same developmental stage, homozygous (Homo, R237H/R237H) mutant rats showed a more pronounced reduction in body weight, weighing approximately 25% of the body weight of WT controls (Figure 1A). Notably, Homo rats exhibited severe lethality, with all individuals succumbing between 36 and 98 days, resulting in a median survival age of 58.6 days (Figure 1B). In contrast, littermates with other genotypes, including Het mutant-null (−/R237H) rats, remained fully viable throughout the observation period. The high mortality observed in Homo rats highlights the critical role of both GFAP mutation and its excess in driving the severe phenotype associated with AxD.

### 2.2. GFAP Overexpression and Solubility Properties in Homozygous AxD Rats

**Figure 2:**
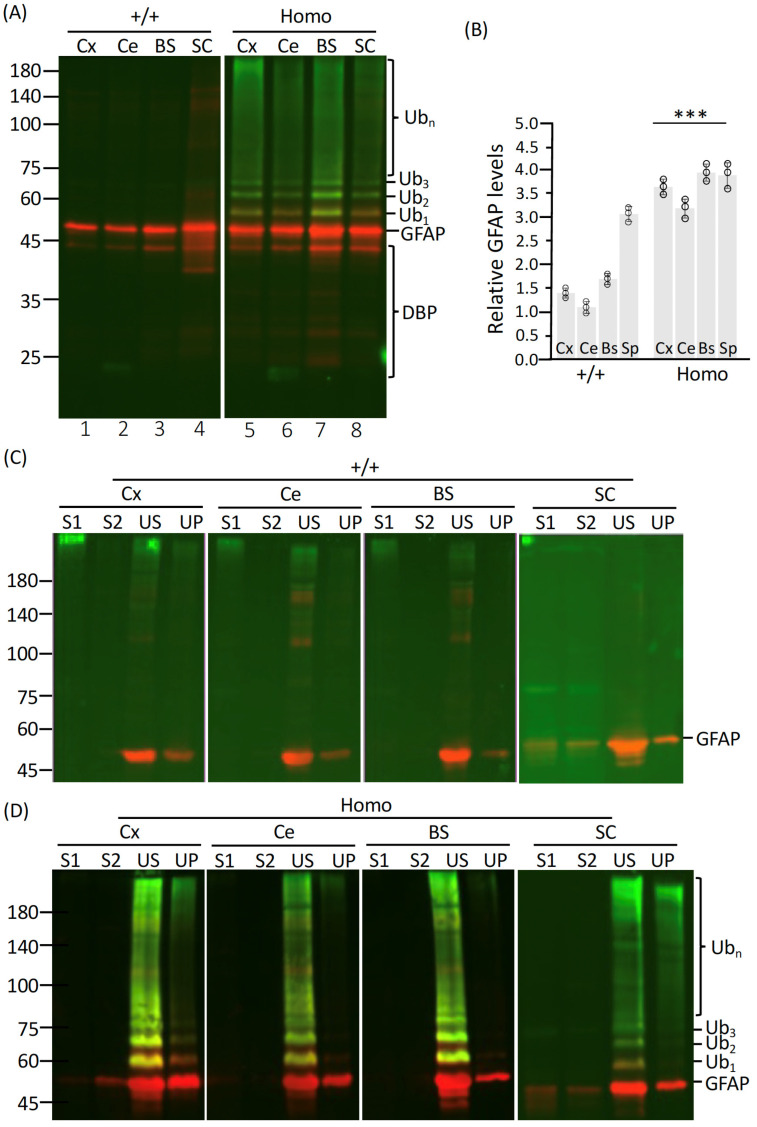
GFAP levels and solubility properties in homozygous AxD rats. (**A**) Total protein lysates were prepared from CNS regions, including cerebral cortex (Cx), cerebellum (Ce), brainstem (BS) and spinal cord (SC) of WT (+/+, lanes 1–4) and Homo (lanes 5–8) rats at 46–58 days of age. Immunoblot analysis was performed using anti-panGFAP (red channel) and anti-ubiquitin (green channel) antibodies. (**B**) Quantification of GFAP levels in tissue lysates relative to WT controls. Each white dot represents a biological replicate (*n* = 3). Data are presented as mean ± SD. Statistical analysis was performed using a two-tailed *t*-test; *** *p* < 0.001. (**C**,**D**) CNS tissues were sequentially extracted into four fractions: S1 (supernatant from detergent extraction), S2 (supernatant from high-salt extraction), US (urea-soluble fraction), and UP (urea-insoluble fraction). Immunoblot analysis was performed on these fractions using anti-GFAP and anti-ubiquitin antibodies. GFAP was predominantly enriched in the urea-soluble (US) fraction across all genotypes, while ubiquitinated GFAP was detected exclusively in Homo rats (**D**). Molecular mass markers (in kDa) are shown on the left, with GFAP and ubiquitinated GFAP species indicated on the right. Ub_1–3_ represent mono-, di-, and tri-ubiquitinated GFAP species, while Ub_n_ indicates polyubiquitinated GFAP. DBP, degradation breakdown product. Protein loading across lanes was confirmed by in-gel staining (Supplementary Figure S2).

To investigate the relationship between GFAP mutation, overexpression, and the observed lethality, we quantified total GFAP levels in various central nervous system (CNS) regions, including the cerebral cortex (Cx), cerebellum (Ce), brainstem (BS), and spinal cord (SC). Immunoblot analysis revealed that Het mutant-null (Supplementary Figure S1A, lanes 5–8) and Homo (Figure 2A, lanes 5–8) rats exhibited significantly elevated GFAP levels across all analyzed CNS regions compared to WT (Figure 2A, lanes 1–4) and Het-null (Supplementary Figure S1A, lanes 1–4) rats. In addition to increased GFAP levels (Figure 2B), Homo rats showed a prominent accumulation of high-molecular-weight ubiquitinated GFAP species (Figure 2A, lanes 5–8), indicative of impaired proteasome-mediated protein degradation. Furthermore, lower-molecular-weight GFAP fragments, likely resulting from proteolytic cleavage by caspases and calpains, were detected mainly in homozygous mutant rats (Figure 2A, lane 7).

To further examine the solubility properties of GFAP, CNS tissues were sequentially extracted using buffers of increasing strength, and the resulting fractions were analyzed by immunoblotting. Full-length GFAP was predominantly enriched in the urea-soluble (US) fraction in both WT (Figure 2C) and Homo (Figure 2D) rats. Similar enrichments were observed in Het-null rats (Supplementary Figure S1C) and Het mutant-null rats (Supplementary Figure S1D). Notably, while high-molecular-weight ubiquitinated GFAP species were absent in WT (Figure 2C) and Het-null (Supplementary Figure S1C) rats, they were exclusively detected in the urea-soluble fraction of Het mutant-null (Supplementary Figure S1D) and Homo (Figure 2D) rats. The accumulation of ubiquitinated GFAP in the urea-soluble fraction of homozygous rats suggests that mutant GFAP is prone to pathological modifications, leading to increased aggregation.

### 2.3. Effects of R237H Mutation on In Vitro Assembly of GFAP

**Figure 3:**
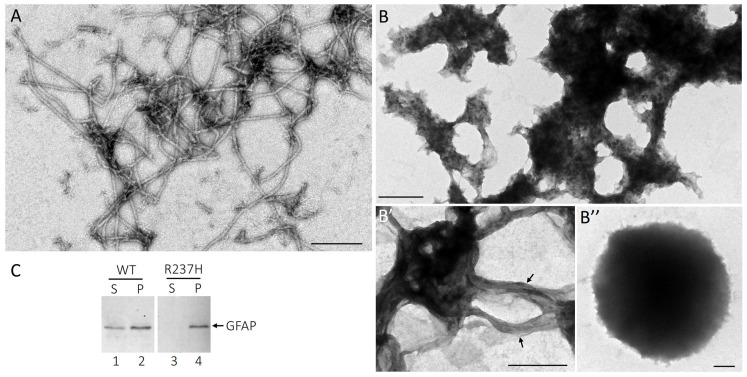
Effect of R237H mutation on GFAP assembly. GFAP purified from spinal cords of WT and homozygous rats were assembled in vitro for 3 h. The assembled filaments were negatively stained and visualized by transmission electron microscopy (TEM). Under these in vitro conditions, wild-type GFAP assembled into typical 10 nm filaments with length of several microns (**A**), whereas R237H GFAP formed irregularly connected fibers with a strong tendency to aggregate (**B**). In regions of less aggregation, sheet-like cable structures ((**B′**), arrows) were observed. At 12 h after assembly, R237H GFAP formed compact, ball-like structures (**B″**). Scale bars, 500 nm. (**C**) Filament assembly efficiency was evaluated using a high-speed sedimentation assay. After 3 h of assembly, samples were subjected to high-speed centrifugation, and the resulting supernatant (S) and pellet (P) fractions were analyzed by SDS-PAGE, followed by Coomassie blue staining. Whereas a small proportion of WT GFAP remained in the supernatant (lane 1), the majority was found in the pellet fraction (lane 2). In contrast, nearly all R237H GFAP was recovered in the pellet fraction (lane 4), with no protein detected in the supernatant fraction (lane 3).

To further explore how the R237H mutation disrupts GFAP assembly and promotes aggregation, we conducted in vitro assembly studies using native GFAP purified from spinal cords of WT and Homo rats. GFAP was first enriched in the urea-soluble fraction using a stepwise extraction protocol (Supplementary Figure S3A,C) and further purified through ion exchange chromatography (Supplementary Figure S3B,D). Under in vitro assembly conditions, WT GFAP readily polymerized into typical 10 nm filaments with lengths extending several microns (Figure 3A). In contrast, R237H GFAP failed to form normal filaments. Instead, it assembled into irregularly connected fiber arrays with a pronounced tendency to aggregate (Figure 3B). In some regions, sheet-like structures were observed (Figure 3B′, arrows), suggesting that individual filaments of mutant GFAP were arrested during the process of lateral fusion. After 12 h of assembly, R237H GFAP further aggregated into compact, ball-like structures (Figure 3B″).

The efficiency of filament assembly was evaluated using a high-speed sedimentation assay. After 3 h of assembly, 26.3% of WT GFAP remained in the supernatant fraction (Figure 3C, lane 1), indicating that the majority of WT GFAP successfully assembled into filaments. In contrast, nearly all R237H GFAP was recovered in the pellet fraction (Figure 3C, lane 4). This increased sedimentation of the R237H mutant suggests a higher propensity for GFAP aggregation, likely due to enhanced inter-filament interactions. Together, these findings demonstrate that the R237H mutation disrupts the structural integrity and normal assembly of GFAP filaments, promoting aberrant aggregation. Such aggregation may act as an initiating event in the pathological cascade underlying AxD.

### 2.4. Effects of Mutant GFAP on Neuronal Intermediate Filament Expression

**Figure 4:**
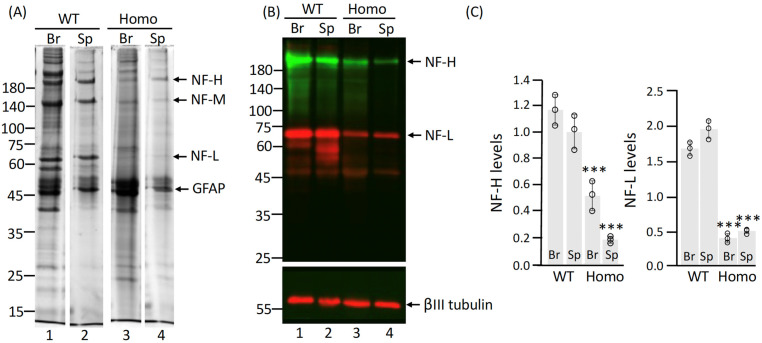
Reduction in neurofilament protein levels in Homo rats. The urea-soluble fractions prepared from the brain (Br) and spinal cord (Sp) of WT and Homo rats were analyzed by SDS-PAGE followed by Coomassie blue-staining (**A**) and immunoblotting (**B**) using antibodies specific to neurofilament-H (NF-H, green channel), neurofilament-L (NF-L, red channel), and βIII tubulin (red channel), which served as a loading control. Molecular mass makers (in KDa) are shown on the left, and the positions of NF-H, NF-M, NF-L and GFAP are indicated on the right. Uncropped images of immunoblots and Coomassie blue-stained gels are provided in Supplementary Figure S4. (**C**) Quantification of NF-H and NF-L levels in the brain (Br) and spinal cord (Sp) of Homo rats relative to WT controls. Each white dot represents a biological replicate (*n* = 3). Data are presented as mean ± SD. Statistical analysis was performed using a two-tailed *t*-test; *** *p* < 0.001.

During the GFAP enrichment procedure, we observed a significant reduction in neurofilament (NF) proteins, including NF-H, NF-M, and NF-L, in the urea-soluble fraction of Homo rats. Coomassie blue-stained gels showed a significant reduction in these proteins in both the brain and spinal cord of mutant samples (Figure 4A, lanes 3 and 4) compared to WT samples (Figure 4A, lanes 1 and 2). This decrease was further confirmed by immunoblot analysis (Figure 4B), with quantification indicating a reduction of approximately 56% in the brain and 82% in the spinal cord relative to wild-type levels (Figure 4C). These results suggest that the R237H mutation not only disrupts the structural integrity and normal assembly of GFAP but also exerts secondary effects on other cytoskeletal components, particularly neuronal IFs. This broader cytoskeletal instability driven by mutant GFAP aggregation may contribute to the neuronal dysfunction and pathological features characteristic of AxD.

### 2.5. Effect of R237H Mutation on GFAP Filament Networks in Astrocytes

**Figure 5:**
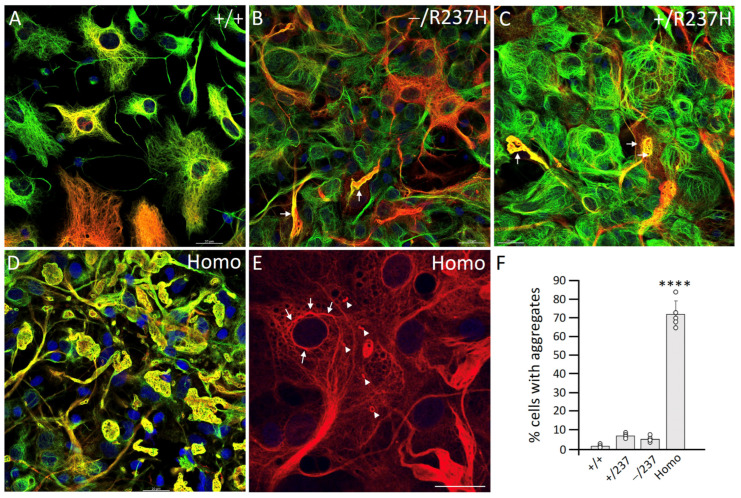
GFAP aggregate formation in astrocytes derived from Homo rats. Primary astrocytes derived from the indicated genotypes were cultured for 14 DIV. Cells were then processed for double-label immunofluorescence microscopy using antibodies against GFAP (red channel) and vimentin (green channel). Merged images are shown, with nuclei visualized by staining with DAPI (blue channel). Scale bar, 20 μm. In WT astrocytes, GFAP formed filamentous intermediate filament (IF) networks that overlapped with vimentin (**A**). In contrast, cytoplasmic aggregates were seen in a small number of GFAP-positive cells in −/R237H ((**B**), arrows) and +/R237H ((**C**), arrows) astrocytes. Note that most Homo astrocytes contained GFAP aggregates that were immunopositive for both GFAP and vimentin (**D**). A representative image shows small aggregates ((**E**), arrowheads) intermingled with perinuclear filament bundles ((**E**), arrows). (**F**) For each genotype, cells on three coverslips were analyzed and their staining patterns were assessed. Each litter was treated as an independent experiment. Quantification of the aggregate-bearing cells from three independent experiments is presented as bar charts, with data shown as mean ± SE. Statistical analysis was performed using a two-tailed *t*-test; **** *p* < 0.0001.

To investigate how GFAP mutation and overexpression affect filament organization and network formation within cells, we examined the assembly and network-forming ability of GFAP in primary astrocytes derived from rat brains. Using immunofluorescence confocal microscopy, we visualized GFAP in primary astrocytes with different genotypes after 14 days in vitro (DIV). In WT astrocytes (Figure 5A) and Het-null astrocytes (Supplementary Figure S5A), GFAP primarily formed well-organized IF networks distributed throughout the cytoplasm, which colocalized with vimentin IFs. In contrast, cytoplasmic aggregates were observed in a small percentage of GFAP-positive cells in −/R237H (Figure 5B, arrows) and +/R237H (Figure 5C, arrows) astrocytes. Strikingly, 72% of GFAP-positive cells in Homo astrocytes contained prominent cytoplasmic aggregates (Figure 5D). These aggregates frequently disrupted the vimentin IF networks, causing them to collapse into large perinuclear aggregates. Additionally, in some Homo astrocytes, GFAP formed perinuclear filament bundles (Figure 5E, arrows) with small aggregates interspersed among these filaments (Figure 5E, arrowheads). This suggests that R237H mutation induces early disruptions in GFAP filament organization, which likely progress to a complete collapse of the IF networks. Quantification of GFAP-positive cells containing aggregates is presented in Figure 5F. Notably, these aggregates appeared metabolically stable, as their prevalence remained consistent even after extended culture for up to 28 DIV. Notably, the proportion of aggregate-containing R237H astrocytes did not significantly change under serum-free conditions (Supplementary Figure S5B), indicating that GFAP aggregate formation is an intrinsic property of the R237H mutation and is independent of culture conditions.

### 2.6. Expression Levels and Solubility Properties of Mutant GFAP

**Figure 6:**
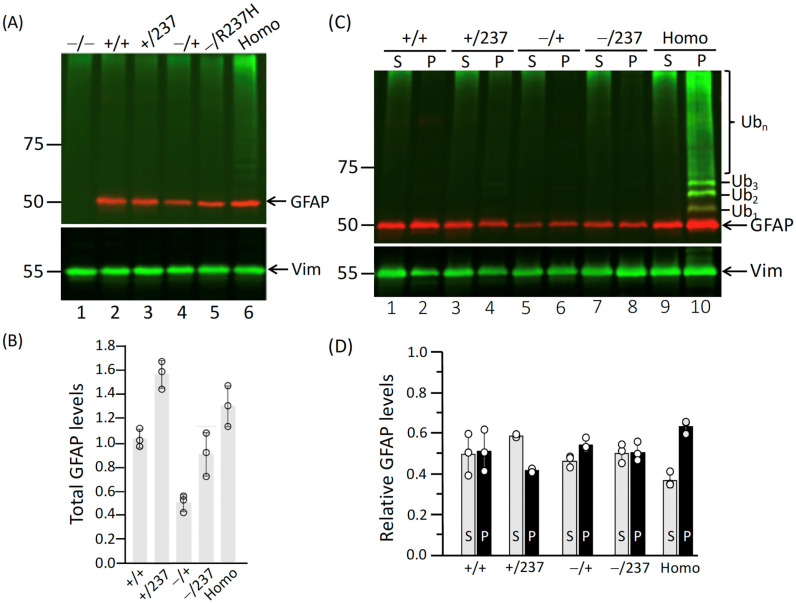
Expression levels and solubility properties of GFAP in primary astrocytes. (**A**) Primary astrocytes derived from rats of the indicated genotypes were extracted with RIPA buffer at 14 DIV. Total protein lysates were analyzed by immunoblotting using antibodies against GFAP (red channel), ubiquitin (green channel), and vimentin (green channel). (**C**) Supernatant (S) and pellet (P) fractions obtained after centrifugation of the RIPA extracts were analyzed by immunoblotting with the same antibodies. Molecular mass markers (in kDa) are shown on the left, while the positions of GFAP, vimentin (Vim), and ubiquitin (Ub) are indicated on the right. A duplicate gel stained with Coomassie blue was used to confirm equal protein loading (Supplementary Figure S6). Notably, ubiquitinated GFAP species were detected exclusively in the pellet fraction of Homo astrocytes ((**C**), lane 10). Quantification of GFAP levels in astrocytes derived from rats with the indicated genotypes is shown in (**B**,**D**). Each white dot represents a biological replicate (*n* = 3). Data are presented as the mean ± SD.

To evaluate GFAP expression at the protein level, total cell lysates were prepared from astrocytes cultured for 14 DIV with the indicated genotypes. Immunoblot analysis using an anti-GFAP antibody showed no GFAP expression in GFAP-null astrocytes (Figure 6A, lane 1). In contrast, astrocytes carrying either WT allele, R237H allele, or a combination of both expressed GFAP at the expected size and in varying levels (Figure 6A, lanes 2–6), confirming that GFAP is expresses from the WT and R237H alleles. Quantitative analysis revealed that Het-mutant and Homo astrocytes exhibited a modest increase in total GFAP levels, ranging from 1.2- to 1.6-fold compared to WT astrocytes (Figure 6B). As expected, Het-null astrocytes (Figure 6A, lane 4) expressed GFAP at approximately half the level observed in WT astrocytes (Figure 6A, lane 1).

Next, we investigated whether the R237H mutation alters the dynamic equilibrium of GFAP between soluble and insoluble pools. To address this, we separated supernatant and pellet fractions from astrocytes cultured for 14 DIV and analyzed GFAP distribution by immunoblotting (Figure 6C). Quantification of the relative distribution of GFAP between the supernatant and pellet fractions is presented in Figure 6D. In WT astrocytes (Figure 6C, lanes 1 and 2) and Het-null astrocytes (Figure 6C, lanes 5 and 6), GFAP was distributed approximately equally between the supernatant and pellet fractions. A similar distribution pattern was observed in Het-mutant (Figure 6C, lanes 3 and 4) and Het mutant-null (Figure 6C, lanes 7 and 8) astrocytes. In contrast, Homo astrocytes exhibited an increase in GFAP levels in the pellet fraction (Figure 6C, lane 10), suggesting enhanced sequestration of GFAP into cytoplasmic aggregates.

### 2.7. AxD Astrocytes Exhibit Stress and Autophagic Responses 

**Figure 7:**
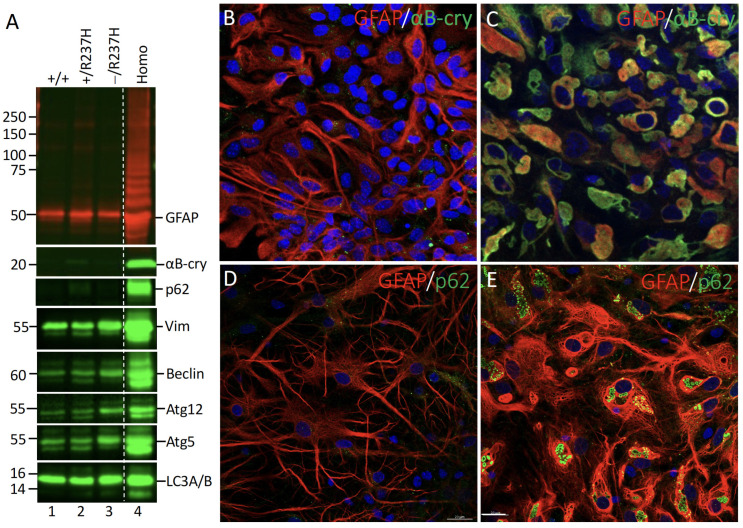
Elevated expression of stress and autophagic markers in Homo astrocytes. (**A**) Primary astrocytes derived from WT (lane 1), Het mutant (lane 2), Het mutant-null (lane 3), and Homo (lane 4) rats were extracted, and the resulting RIPA-insoluble fractions were analyzed by immunoblotting using antibodies specific to the indicated proteins. All protein signals were displayed in the green channel, except for GFAP, which was shown in the red channel. Molecular mass markers (in kDa) are displayed on the left, while the positions of analyzed proteins are indicated on the right. A duplicate gel stained with Coomassie blue was used to confirm equal protein loading (Supplementary Figure S7A). Uncropped images of the immunoblots are provided in Supplementary Figure S7B–H. The dashed line in (**A**) indicates that lanes were run on the same gel but were noncontiguous. WT (**B**,**D**) and Homo (**C**,**E**) astrocytes cultured for 14 DIV were processed for double-label immunofluorescence microscopy. Cells were immunostained with anti-GFAP antibody ((**B**–**E**), red channel) and either anti-αB-crystallin ((**B**,**C**), green channel) or anti-p62 antibody ((**D**,**E**), green channel). Nuclei were visualized by DAPI staining (blue channel) and merged images are shown. Scale bar, 20 μm. GFAP aggregates in homozygous R237H astrocytes were immunopositive for both αB-cry (**C**) and p62 (**E**).

The shift in GFAP distribution was accompanied by a marked elevation in vimentin and the accumulation of high-molecular-weight ubiquitinated GFAP species (Figure 7A, lane 4), which point to impaired proteasome-mediated degradation. Additionally, proteins involve in cellular stress and autophagic responses, such as αB-crystallin and p62, were also elevated in the pellet fraction of Homo astrocytes (Figure 7A, lane 4). Other key markers of autophagic activity, including beclin, Atg12, Atg5, and LC3A/B, were similarly increased (Figure 7A, lane 4), further supporting the activation of autophagic pathways in response to GFAP aggregation. To further investigate whether these stress-related proteins are associated with GFAP aggregates in Homo astrocytes, we performed double-label immunofluorescence microscopy. Notably, both αB-crystallin (Figure 7C) and p62 (Figure 7E) colocalized with the GFAP-containing aggregates in Homo astrocytes, confirming their associations with pathological inclusions. In contrast, WT astrocytes exhibited significant lower levels of αB-crystallin (Figure 7B) and p62 (Figure 7D), which were diffusely distributed throughout the cytoplasm without evidence of aggregation. These results suggest that the aggregates formed in Homo astrocytes share key features characteristic of Rosenthal fibers, a pathological hallmark of AxD.

### 2.8. Non-Cell Autonomous Effects of AxD Astrocytes on Neurons

**Figure 8:**
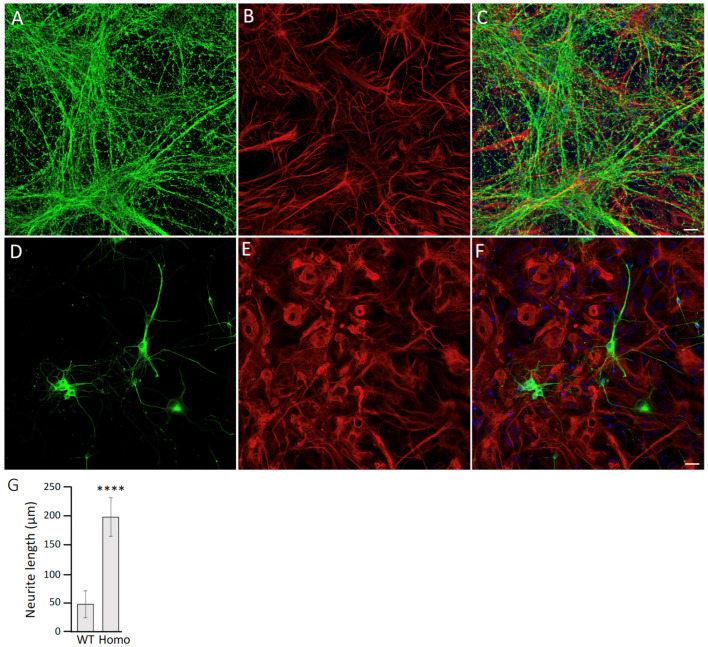
Expression of mutant GFAP in Homo astrocytes alters neurite morphology. Primary neurons cocultured with either WT (**B**) or Homo (**E**) astrocytes were immunostained with antibodies against βIII tubulin ((**A**,**D**), green channel) to visualize neurons and GFAP ((**B**,**E**), red channel) to label astrocytes. Merged images are shown (**C**,**F**), with nuclei visualized by DAPI staining (blue channel). Bar, 20 μm. Neurite length of neurons cocultured with either WT or Homo astrocytes were quantified and are presented as bar chars (**G**). Data are shown as mean ± SD. Statistical analysis was performed using a two-tailed *t*-test, with statistical significance between WT and R237H astrocytes indicated by **** *p* < 0.0001.

Since a key function of astrocytes is to support neurons, we next investigated whether the accumulation of R237H GFAP in astrocytes exerts non-cell-autonomous effects on neurons using astrocyte–neuron co-cultures. Rat E18 neurons were seeded onto primary astrocytes at 14 DIV and allowed to mature for an additional 10 days. The cocultures were then fixed and immunostained with antibodies against βIII tubulin, a neuronal cytoskeletal protein, to assess neuronal morphology. Neurons cocultured with WT astrocytes (Figure 8A) exhibited significantly longer neurite lengths compared to those cocultured with Homo astrocytes (Figure 8D). Specifically, the neurite lengths of neurons cocultured with Homo astrocytes were reduced by 76% compared to those of neurons cocultured with WT astrocytes (Figure 8G). This pronounced reduction in neurite outgrowth suggests that the accumulation of R237H GFAP in astrocytes disrupts their ability to support normal neuronal development and morphology, potentially contributing to the neurodegenerative phenotype observed in AxD.

## 3. Discussion

### 3.1. Qualitative Defects and Quantitative Overload of Mutant GFAP in AxD Pathology

Alexander disease (AxD) is predominantly associated with genetically dominant mutations in the GFAP gene, which are believed to confer a toxic gain of function. However, recent studies have identified rare cases of AxD with recessive inheritance [6,7,8]. Intriguingly, these mutations were previously classified as dominant in other AxD patients [24,25,26], suggesting that the observed inheritance patterns may be better explained by dominant inheritance combined with gene dosage effects. This complexity highlights the need to investigate both the nature of GFAP mutations and their expression levels to fully understand AxD pathogenesis.

In the disease setting where patients with AxD are typically heterozygous, the GFAP pool consists of both wild-type and mutant proteins. Distinguishing between these two forms of GFAP is critical for understanding the specific effects of the mutation. However, separating wild-type GFAP from mutant protein is technically challenging, even with advanced methods such as high-resolution column chromatography [27]. To overcome this limitation, we generated rat models that exclusively express the mutant form of GFAP at different levels. This approach allows us to more precisely investigate the specific effects of the AxD mutation and its expression levels on GFAP aggregation and astrocyte dysfunction, setting the stage for deeper insights into AxD mechanisms.

Early-onset AxD is often associated with failure to thrive, a clinical feature characterized by poor weight gain and growth deficits [2]. Consistent with this observation, reduced body weight is a well-documented phenotype in AxD rodent models [16,23,28]. In our study, Homo rats exhibited normal early postnatal development but showed significant growth impairment after weaning, failing to reach the size of their WT littermates. This failure to thrive likely reflects underlying disease mechanisms, including astrocyte dysfunction and its systemic effects, which compromise overall health and development.

Several factors may contribute to these weight deficits, including oropharyngeal dysphagia—a common AxD symptom that impairs swallowing and causes feeding difficulties [2,29]—and frequent emesis, which can exacerbate malnutrition [30]. Additionally, developmental defects in homozygous rats may arise from neuroendocrine dysfunction, potentially due to impaired astrocytic support of hypothalamic neurons. Given the critical role of the hypothalamus in regulating hormonal balance, appetite, energy metabolism, and stress responses, its dysfunction could have far-reaching systemic consequences. While these factors were not directly assessed in our study, they represent important avenues for future research using this model.

Beyond growth deficits, some surviving Homo rats developed hindlimb monoplegia between 6 and 8 weeks of age, and we observed seizure-like episodes in two animals during terminal events. Seizures, a hallmark of early-onset AxD, may contribute to the increased mortality observed in Homo rats. However, the prevalence and impact of seizures in this severe model remain understudied. Future investigations should incorporate electroencephalogram (EEG) monitoring to evaluate the role of seizure activity in growth deficits and early mortality in Homo rats. Understanding the link between seizures and disease progression could provide critical insights into AxD pathophysiology and inform therapeutic strategies.

The toxicity of GFAP excess in AxD is well documented, as overexpression of even wild-type human GFAP in mouse models induces severe pathology, including Rosenthal fiber formation and premature death [16,20]. These models underscore the inherent toxicity of GFAP excess [17]. However, focusing solely on overexpression oversimplifies the complex mechanisms underlying AxD, as the presence of mutant GFAP introduces additional pathogenic dimensions. Our in vitro studies show that the R237H mutant GFAP remains assembly competent but forms abnormal filaments that aggregate over time, suggesting that the mutation itself is the initiating event in pathogenesis, preceding any changes in expression levels. This is further supported by our observation that Het mutant-null rats, without excessive total GFAP levels, still develop pathology due to the qualitative defect of the mutant protein.

Moreover, our study reveals a clear gene dosage effect, where disease severity and lifespan correlate with the number of mutant GFAP alleles. Homo rats express higher levels of mutant GFAP and exhibit more severe pathology and earlier mortality compared to Het mutant-null rats (−/R237H). This indicates that quantitative overload of mutant GFAP exacerbates disease progression, amplifying the toxic effects of the mutation. These findings emphasize the dual role of qualitative defects of mutant GFAP and its quantitative overload by gene dosage in driving AxD pathology.

### 3.2. Comparison of GFAP and Desmin Variants

Both GFAP and desmin (DES) are type III IF proteins that share significant structural and functional similarities, including a conserved central rod domain essential for filament assembly and higher-order network formation. Mutations in both genes are associated with protein aggregation and disease pathogenesis, though they occur in distinct cellular contexts—astrocytes for GFAP and muscle cells for DES. Despite these differences, the molecular defects caused by mutations in GFAP and DES exhibit notable parallels.

Our previous studies on various GFAP variants, including R239C and R239H, demonstrated that amino acid substitutions in the rod domain have differential effects on filament assembly and network formation [18]. For instance, the R239H mutation disrupts higher-order filament interactions, leading to aggregation and the formation of Rosenthal fibers, a hallmark of AxD [27]. Similarly, mutations in DES interfere with filament assembly at various stages, as shown in studies of desmin mutations and their effects on filament formation [31]. Mutant desmin can co-aggregate with wild-type desmin, consistent with a dominant-negative mechanism, which is analogous to the co-polymerization and aggregation behavior observed in GFAP mutations. Both cases highlight the disruption of cytoskeletal integrity—within astrocytes for GFAP and muscle cells for DES.

In addition to pathogenic mutations, post-translational modifications (PTMs) such as phosphorylation and ubiquitination have been shown to influence GFAP filament assembly and promote aggregation [19]. However, prior in vitro studies used recombinant GFAP produced in bacteria, which lack eukaryotic PTMs, limiting the ability to fully assess their role in filament assembly. In contrast, DES mutations are known to affect filament assembly in the presence of PTMs, as this muscle-specific IF protein undergoes a unique set of PTMs [32]. These modifications appear to modulate the aggregation and co-polymerization of mutant and wild-type desmin filaments, as observed in cardiomyopathy-associated DES variants [33].

In this study, we provide the first demonstration of assembly defects using native GFAP purified from the spinal cords of R237H homozygous AxD model rats. This physiologically relevant approach allows us to better understand the impact of mutant GFAP on filament assembly and aggregation, overcoming the limitations of recombinant protein studies that lack eukaryotic PTMs. However, our understanding of the higher-order interactions within the GFAP filament remains constrained due to the absence of high-resolution structural data. A detailed 3D model of the glial filament is essential for elucidating the molecular mechanisms underlying GFAP mutation-induced defects. Similarly, while studies have explored the effects of DES mutations on filament assembly [31,34], the structural details of desmin filaments remain incomplete, limiting the extent to which molecular consequences of mutations in GFAP and DES can be fully compared. Further structural studies of both GFAP and DES filaments are critical to advancing our understanding of their shared and unique molecular pathomechanisms.

### 3.3. Discrepancy of GFAP Solubility Between Rat AxD Model and Human AxD Patients

The detection of ubiquitinated GFAP species exclusively in the Het mutant-null and Homo rats suggests mutant GFAP is prone to pathological modifications, leading to aggregation. Sequential extraction of rat CNS tissues revealed that GFAP was predominantly enriched in the urea-soluble fraction across all genotypes. However, when using the same extraction protocol to analyze human AxD brain tissues, GFAP and its modified forms were enriched predominantly in the urea-insoluble fraction [19]. This discrepancy highlights important differences between human and rat AxD, which are likely influenced by tissue handling, storage conditions, and inherent biological and pathological differences.

One primary factor contributing to this difference is the impact of long-term storage on human tissues. Human AxD brain samples are often stored in freezers for many years before analysis. Prolonged freezing, even under optimal conditions, can lead to protein degradation, aggregation, or cross-linking, potentially rendering GFAP and its modified forms less soluble and resulting in their enrichment in the urea-insoluble fraction. Additionally, factors such as post-mortem interval (PMI) before freezing and storage conditions could exacerbate protein insolubility, particularly in AxD, where GFAP aggregation into Rosenthal fibers is a hallmark feature. In contrast, the AxD rat tissues used in this study were freshly collected and immediately processed for biochemical extraction. Fresh tissues are less susceptible to the protein modifications or structural changes associated with long-term storage, which likely preserves GFAP in a more soluble state, leading to its enrichment in the urea-soluble fraction. The absence of prolonged post-mortem changes or storage-related degradation in rat tissues may minimize the formation of stable, insoluble aggregates during the extraction process. This discrepancy underscores the value of using fresh tissues whenever possible to capture the dynamic biochemical properties of pathological proteins like GFAP in neurodegenerative diseases.

Beyond storage conditions, biological and pathological differences between human and rat tissues may also contribute to the observed solubility discrepancy. It is important to recognize that human patients typically have more extensive pathology than any of the animal models to date. Human AxD tissues often represent end-stage disease with advanced Rosenthal fiber formation and chronic pathological changes, potentially resulting in more stable, insoluble aggregates. In comparison, the rat model, even in severe homozygous mutant cases, may reflect an earlier or less advanced stage of aggregation, leading to more soluble GFAP forms. In addition, differences in specific GFAP mutations, genetic backgrounds, post-translational modifications and the duration of disease progression between humans and rats could influence the biochemical state of GFAP aggregates and solubility profiles.

Future studies could address these differences by optimizing storage conditions to directly assess the impact of storage time on GFAP quality. Moreover, standardizing post-mortem intervals and freeze–thaw cycles, as well as investigating the correlation between disease stage, Rosenthal fiber load and solubility properties, will be critical for refining animal models to more accurately reflect human AxD pathology.

### 3.4. Stress and Autophagic Responses in AxD Astrocytes

At the cellular level, the R237H mutation exerts toxic effects by disrupting filament network formation, promoting aggregate accumulation, and increasing GFAP ubiquitination. The presence of ubiquitinated GFAP species in astrocytes of the Homo rats further supports that mutant GFAP is prone to pathological modifications, which facilitate its aggregation [19]. In Homo astrocytes, 72% of GFAP-positive cells exhibit aggregates, compared to less than 10% in Het mutant and Het mutant-null astrocytes. This striking contrast highlights a gene dosage effect, where elevated levels of mutant GFAP overwhelm the cellular proteostasis machinery, leading to widespread aggregation. Notably, Homo astrocytes show an approximately 1.6-fold increase in GFAP levels compared to WT astrocytes, suggesting that the threshold for GFAP toxicity is significantly lower when mutant protein is present. Supporting this observation, GFAP aggregates form in 30% of transgenic (Tg) astrocytes when total GFAP levels are 3–5 times higher than in WT astrocytes [35]. Further evidence comes from the analysis of transgenic mouse lines expressing varying levels of the AxD-causing human R239H mutant GFAP [11]. This analysis revealed that the expression of mutant GFAP alone does not trigger Rosenthal fiber formation; instead, aggregates emerge only when total GFAP levels increase by 2.7% to 30%. These findings reinforce the concept of a toxic threshold, where the combination of qualitative defects in mutant GFAP and quantitative overload due to increased expression synergistically drives pathological aggregation.

GFAP aggregation triggers significant cellular stress responses in AxD astrocytes. The small heat shock protein αB-crystallin (CryαB), a molecular chaperone essential for cytoskeletal integrity [36], is upregulated under stress and observed in various CNS inclusion body disorders [37]. CryαB facilitates GFAP oligomer disassembly and alleviates proteasome inhibition, playing a protective role [38]. In Homo rats, we observed a significant increase in CryαB expression in astrocytes, consistent with prior AxD rodent studies [14,23,39]. However, despite this upregulation, severe phenotypes and early lethality persist, indicating that CryαB’s protective capacity is insufficient against mutant GFAP toxicity. Notably, CryαB shifts to the insoluble fraction, suggesting increased interaction with GFAP aggregates, which may impair its chaperone function and stabilize aggregates rather than clear them, potentially contributing to early Rosenthal fiber formation [40].

The stress response in AxD astrocytes is further linked to the role of p62 [41], a multifunctional adaptor protein critical for proteostasis via the ubiquitin–proteasome system (UPS) and autophagy. In Homo astrocytes, ubiquitinated GFAP indicates active UPS degradation attempts, but the proteasome appears overwhelmed by aggregated GFAP. Consequently, p62 is recruited to facilitate autophagic clearance, yet its accumulation—along with elevated levels of autophagic markers like LC3A/B, Atg5, and Atg12—suggests impaired autophagy. This aligns with p62 accumulation in Rosenthal fibers in human AxD patients [42]. The failure of these mechanisms to restore proteostasis, evidenced by CryαB sequestration and p62 buildup, underscores their dual protective and pathological roles in AxD progression.

### 3.5. Non-Cell Autonomous Effect of Astrocyte Dysfunction on Neurons

Astrocyte dysfunction in AxD has profound non-cell-autonomous effects on neurons, as astrocytes are vital for supporting neuronal health through synapse regulation, synaptic function, and metabolic homeostasis. Our data show that Homo rats exhibit significantly reduced neurofilament proteins (NF-H, NF-M, NF-L) in the brain and spinal cord, a finding mirrored in transgenic mice overexpressing normal human GFAP [20], suggesting that elevated GFAP levels alone impair neuronal integrity. Furthermore, primary neurons cocultured with homozygous mutant astrocytes display reduced neurite length, indicating that astrocyte dysfunction driven by mutant GFAP directly compromises neuronal health by impairing neurite outgrowth and maintenance. These observations highlight the critical role of astrocyte–neuron crosstalk in AxD pathogenesis and support the classification of AxD as a primary astrogliopathy, where astrocyte dysfunction drives secondary neuronal damage and broader CNS pathology.

The mechanisms underlying reduced neurofilament levels due to mutant GFAP expression and aggregation remain unclear. However, studies using a Drosophila model suggest that glial-derived nitric oxide (NO) may mediate astrocyte-induced neurodegeneration [43]. High NO levels generate reactive nitrogen species (RNS), such as peroxynitrite (ONOO^−^), causing oxidative and nitrosative stress that damages neuronal lipids, proteins, and DNA, potentially exacerbating AxD neurodegeneration [44]. While the role of NO in this context awaits experimental validation, our findings point to a non-cell-autonomous mechanism of neuronal damage, emphasizing the need to target astrocyte–neuron interactions in therapeutic approaches.

### 3.6. Concluding Remarks and Future Perspectives

In summary, our study elucidates the multifaceted pathogenesis of AxD, driven by the intricate interplay of genetic dominance, qualitative defects in mutant GFAP, and quantitative overload due to gene dosage effects. These factors converge to cause GFAP aggregation, disrupt astrocyte function, and trigger cellular stress responses, forming the core of AxD pathology. Using the rat AxD models, we reveal that qualitative defects in mutant GFAP initiate assembly defect and filament aggregation, while gene dosage effects amplify toxicity, as higher mutant GFAP levels correlate with increased aggregate formation, severe pathology, and reduced survival. Furthermore, the insufficiency of protective mechanisms, such as CryαB chaperone activity and p62-mediated autophagy, underscores the cellular inability to mitigate GFAP toxicity, exacerbating astrocyte dysfunction. This primary astrogliopathy extends beyond astrocytes, exerting profound non-cell-autonomous effects on neurons, evidenced by reduced neurofilament levels and impaired neurite outgrowth, which contribute to neurodegeneration and clinical manifestations like growth deficits and seizures. Looking forward, future research must unravel mechanisms such as glial-derived NO-mediated neuronal toxicity and the role of seizures in early mortality to inform future therapies. By targeting both astrocyte dysfunction and its downstream neuronal consequences, these insights pave the way for developing precise interventions to halt or reverse the devastating progression of AxD.

## 4. Materials and Methods

### 4.1. Materials

The general chemicals used in this study were of analytical grade or higher, sourced from Sigma-Aldrich (St. Louis, MO, USA). Specific chemicals such as protease inhibitors, including leupeptin, aprotinin, N-acetyl-leu-leu-norleucinal (ALLN), and phenylmethylsulfonyl fluoride (PMSF), were also obtained from Sigma-Aldrich. An 8 M urea stock solution was prepared using analytical-grade urea (Sigma-Aldrich, Cat. #U5128) and deionized with a mixed-bed ion-exchange resin (Amberlyte, Sigma-Aldrich, Cat. #MB-6113). Buffers were prepared as fresh as possible with ultrapure water (Millipore Milli-Q system, Burlington, MA, USA). Ionic and non-ionic detergents, including Nonidet P-40 (NP-40), Triton X-100, Tween 20, and sodium deoxycholate, were purchased from Sigma-Aldrich. Reagents for protein electrophoresis, such as 30% Acrylamide-Bis solution, N,N,N′,N′-tetramethylethylenediamine (TEMED), β-mercaptoethanol, ammonium persulfate (APS), sodium dodecyl sulfate (SDS), and Coomassie brilliant blue R250, were obtained from Bio-Rad (Hercules, CA, USA). Nitrocellulose membranes for immunoblotting were sourced from Pall Corporation (Port Washington, NY, USA), and protein concentrations were determined using the bicinchoninic acid (BCA) assay kit (Thermo Fisher Scientific, Waltham, MA, USA). Columns used for GFAP purification were purchased from Cytiva (Marlborough, MA, USA).

Reagents for cell culture, including Hank’s Balanced Salt Solution (HBSS), Dulbecco’s Modified Eagle Medium (DMEM), Minimum Essential Medium (MEM), and Phosphate-Buffered Saline (PBS), were of molecular biology grade and purchased from Thermo Fisher Scientific (Waltham, MA, USA). Cell culture media were supplemented with One Shot fetal bovine serum (FBS, Thermo Fisher, Cat. #A5209401, endotoxin < 10 EU/mL) and 1% penicillin–streptomycin (Gibco, Grand Island, NY, USA, Cat. #15140-122). Enzymes such as 0.25% trypsin solution (Gibco, Cat. #25200056) and DNase I (Sigma-Aldrich, Cat. #DN25, ≥90% purity), used for enzymatic dissociation of brain tissues, were prepared fresh or thawed immediately before use under sterile conditions. Poly-L-lysine hydrobromide (Sigma-Aldrich, Cat. #P4707, molecular weight 70,000–150,000) was used to coat culture dishes. Dissociated cell suspensions were filtered using 70 µm nylon cell strainers (Corning, Corning, NY, USA, Cat. #431751). Culture dishes and plates were sourced from Greiner Bio-One (Kremsmünster, Austria). DAPI (4′,6-diamidino-2-phenylindole, Sigma-Aldrich, Cat. #D8417) was used for nuclear staining of cultured cells.

### 4.2. Rat Model of AxD

All animal experiments were conducted in accordance with the guidelines outlined in the Agriculture Guidebook for the Care and Use of Laboratory Animals and were approved by the Institutional Animal Care and Use Committee of the College of Life Sciences and Medicine at National Tsing Hua University (NTHU IACUC Approval Nos. 109088 and 111060). The *gfap* knockout (−/−) and heterozygous R237H knock-in (+/R237H) rats used in this study were generously provided by Dr. Tracy Hagemann (Waisman Center, University of Wisconsin-Madison). These models were generated using CRISPR-Cas9-mediated genome editing to introduce a *gfap*-null mutation and the R237H mutation into the endogenous rat *gfap* gene [23]. The R237H mutation is orthologous to the R239H mutation commonly associated with early-onset AxD in humans. Rats carrying the desired mutations were genotyped via polymerase chain reaction (PCR) analysis of DNA extracted from tail biopsies, using primers flanking exon 4. Unless otherwise stated, male and female rats aged 8 to 12 weeks were used in all experiments. Experimental groups consisted of Het mutant-null (−/R237H) and Homo (R237H/R237H) rats, with sex- and age-matched wild-type (+/+) and Het null (−/+) littermates serving as controls. The genotype of all animals was confirmed by PCR prior to their inclusion in the study.

### 4.3. GFAP Enrichment by Sequential Extraction

GFAP-enriched fractions were isolated from selected brain regions using a previously established fractionation protocol [27]. Briefly, brain tissues were homogenized with TEN buffer (10 mM Tris-HCl, pH 7.4, 100 mM NaCl, and 5 mM EDTA) and centrifuged at 76,000× *g* for 20 min at 4 °C. The resulting pellet was sequentially extracted using the following buffers: (1) Triton X-100 buffer: (1% (*v*/*v*) Triton X-100 in TEN buffer; (2) sucrose buffer: 0.85 M sucrose and 0.5% (*v*/*v*) Triton X-100 in TEN buffer; (3) high-salt buffer (1.5 M KCl and 0.5% (*v*/*v*) Triton X-100 in TEN buffer). After each extraction, the samples were centrifuged at 76,000× *g* for 20 min at 4 °C to collect the pellet. Following the final extraction step, the pellet was resuspended in 8 M urea in TEN buffer and incubated overnight at 4 °C with continuous rotation. The samples were then centrifuged, and the supernatant was collected as urea-soluble fraction. The protein concentration of urea-soluble fraction was quantified using a bicinchoninic acid (BCA) assay. The protein samples were then analyzed by immunoblotting.

### 4.4. Immunoblotting

Immunoblotting was conducted using a wet electrophoretic transfer system (Bio-Rad) as described previously [19]. After protein transfer onto nitrocellulose membranes (Pall Life Sciences, Portsmouth, UK), membranes were blocked with 3% (*w*/*v*) bovine serum albumin (BSA) prepared in Tris-buffered saline (TBS: 20 mM Tris-HCl, pH 7.4, 150 mM NaCl) with 0.1% (*v*/*v*) Tween (TBST) for 1 h at room temperature. Following blocking, the membranes were washed with TBST and incubated overnight at 4 °C with primary antibodies (Table 1). The next day, membranes were washed again with TBST and incubated for 1 h at room with horseradish peroxidase (HRP)-conjugated secondary antibodies (anti-mouse, anti-rabbit, or anti-rat). Protein signals were detected using Enhanced Chemiluminescence (ECL) substrate (Western Lightning, PerkinElmer Life Sciences, Shelton, CT, USA) and visualized using the ChemiDoc MP Imaging System (Bio-Rad). For multiplex fluorescent immunoblotting, goat anti-mouse and anti-rabbit IgG conjugated with StarBright Blue 520 or StarBright Blue 700 (Bio-Rad) were used as secondary antibodies. Non-saturated exposures of immunoblots were analyzed and quantified using ImageLab Software (Bio-Rad). Additionally, in-gel staining with trichloroethanol (Sigma-Aldrich) was performed as needed to visualize total protein profiles.

### 4.5. Purification of R237H GFAP

GFAP from the urea-soluble fraction was purified using ion-exchange chromatography, with the NGC chromatography system (BioRad) equipped with a Q-Sepharose column (Cytiva, Marlborough, MA, USA). The column was pre-equilibrated with Q column buffer (6 M urea, 10 mM Tris-HCl (pH 8.0), 5 mM EDTA, and 14.4 mM β-mercaptoethanol). GFAP was eluted from the column using a linear gradient of 0–0.5 M NaCl in the Q column buffer over 1 h at a flow rate of 1 mL/min. The GFAP-enriched fractions were pooled and dialyzed against S column buffer (6 M urea, 20 mM MES (pH 6.0), and 14.4 mM β-mercaptoethanol). The dialyzed sample was then applied to a HiRes S column (Cytiva) pre-equilibrated with S column buffer. After washing the column, GFAP was eluted using a linear gradient of 0–1 M NaCl in S column buffer. Fractions collected from both purification steps were analyzed by SDS-PAGE, followed by Coomassie blue staining to confirm the presence of purified GFAP. Fractions containing purified GFAP were pooled and the protein was stored at −80 °C. The concentration of purified GFAP was quantified using a bicinchoninic acid assay (Thermo Fisher Scientific) with bovine serum albumin (BSA) as the standard.

### 4.6. In Vitro Assembly and Sedimentation Assay

The in vitro assembly of GFAP and sedimentation assay were performed as described previously [45]. Briefly, purified GFAP at a concentration of 0.1 mg/mL was prepared in a low-ionic-strength buffer (10 mM Tris-HCl (pH 8.0), 5 mM EDTA, 1 mM EGTA, and 14.4 mM β-mercaptoethanol) containing 6 M urea. The protein sample was then dialyzed stepwise into the same buffer without urea. GFAP intermediate filament assembly was initiated by adding a 20-fold concentrated assembly buffer to achieve a final concentration of 100 mM imidazole-HCl, pH 6.8, 0.5 mM DTT. The reaction mixture was incubated at 37 °C to promote filament assembly.

To visualize the assembled GFAP, glow-discharged, carbon-coated copper grids (Ted Pella Inc., Redding, CA, USA) were placed on a 20 μL drop of the sample and left to absorb for 1 min at room temperature. Excess liquid was removed by wicking with filter paper, and the grid was rinsed twice with distilled water. The samples were then stained with 1% (*w*/*v*) uranyl acetate (Electron Microscopy Sciences, Hatfield, PA, USA) for 1 min, followed by air drying at room temperature for 5 min. The grids were examined using a transmission electron microscope (HT-7700, Hitachi High-Tech, Tokyo, Japan) operating in high-resolution mode at 100 kV. Images were captured using a CCD camera and further processed with Adobe Photoshop CC (Adobe Systems, San Jose, CA, USA).

The efficiency of GFAP assembly was evaluated using sedimentation assay as described previously [46]. In brief, the assembly mixture was layered onto a 0.85 M sucrose cushion prepared in assembly buffer and centrifuged at 80,000× *g* for 20 min at 20 °C. The supernatant and pellet fractions were collected separately and analyzed by SDS-PAGE, followed by Coomassie blue staining. The relative distribution of GFAP in the pellet and supernatant fractions was analyzed using the ChemiDoc MP Imaging System (Bio-Rad, Hercules, CA, USA) and quantified by the ImageLab Software (v. 6.1, Bio-Rad, Hercules, CA, USA).

### 4.7. Astrocyte-Enriched Primary Cultures

Astrocyte-enriched primary cultures were prepared from postnatal day 2–3 pups of the indicated genotypes using standard protocols as previously described [47]. Briefly, cerebral cortices were dissected in Hank’s Balanced Salt Solution (HBSS) and digested with 0.25% (*w*/*v*) trypsin at 37 °C for 15 min. To further aid tissue dissociation, DNase I (Sigma-Aldrich) was added, and the tissue was incubated for an additional 5 min. Following enzyme digestion, the cortices were mechanically dissociated by gentle trituration using a Pasteur pipette. Dissociated cells were collected by centrifugation at 1000× *g* for 5 min and subsequently resuspended in plating medium. The plating medium consists of minimal essential medium (MEM) supplemented with 5% (*v*/*v*) horse serum, 5% (*v*/*v*) fetal bovine serum, 100 U/mL penicillin, and 100 μg/mL streptomycin. The cell suspension was filtered through a 70 μm nylon mesh (Greiner Bio-One) to remove debris and clumps before plating onto poly-D-lysine-coated plates or dishes at a density of 5 × 10^4^ cells/cm^2^. Cultures were maintained in a humidified incubator at 37 °C with 95% (*v*/*v*) air and 5% (*v*/*v*) CO_2_ for 10 to 14 days in vitro (DIV). The medium was replaced every 2 days to support cell growth and viability.

### 4.8. Immunofluorescence Microscopy

Cells were fixed in 4% (*v*/*v*) paraformaldehyde (Electron Microscopy Sciences) prepared in PBS for 15 min at room temperature. Following fixation, cells were permeabilized with 0.2% (*w*/*v*) Triton X-100 (Sigma-Aldrich) in PBS for 5 min and subsequently blocked with 10% (*v*/*v*) normal goat serum (Jackson ImmunoResearch Laboratories, West Grove, PA, USA) in PBS for 1 h at room temperature. After blocking, cells were washed with PBS and incubated with primary antibodies (Table 1) for at least 1 h at room temperature. After washing off unbound primary antibodies, cells were incubated with secondary antibodies conjugated to Alexa Fluor 488 or Alexa Fluor 594 (Thermo Fisher Scientific) for 1 h at room temperature. Nuclei were counterstained with 4,6-diamidino-2-phenylindole (DAPI) to visualize nuclear DNA. Immunostained cells were imaged using a Zeiss LSM800 laser scanning confocal microscope (Carl Zeiss, Oberkochen, Germany) equipped with 20× (0.7 NA) Plan-Neofluar and 40× (1.3 NA) Apochromat objective lenses. Images were acquired as 0.5 μm optical sections using Zen software (v2.3) and subsequently processed and analyzed with Adobe Photoshop CC (Adobe Systems). For quantification of GFAP aggregation, random fields from at least three coverslips were imaged. The percentage of cells displaying GFAP-positive aggregates was determined by visual assessment of the immunofluorescence images.

### 4.9. Subcellular Fractionation

Cells were cultured in 6-well plates at a density of 5 × 10^5^ cells per well and grown for the indicated number of days in vitro. At the desired time point, the cells were rinsed with PBS and homogenized in ice-cold RIPA buffer (50 mM Tris, pH 8.0, 150 mM NaCl, 1% NP-40, 5 mM EDTA, 0.5% sodium deoxycholate, and 0.1% SDS) supplemented with a protease inhibitor cocktail. A small aliquot of the homogenate was set aside as the total cell lysates. The remaining homogenates were centrifuged at 17,000× *g* for 15 min at 4 °C. The supernatant obtained after centrifugation was designated as the RIPA-soluble fraction and stored for subsequent analysis. The pellet, representing the RIPA-insoluble fraction, was resuspended in TES buffer (20 mM Tris-HCl, pH 7.4, 5 mM EDTA, 1% (*w*/*v*) SDS) containing protease inhibitors and sonicated to ensure complete solubilization. Protein concentrations of both the RIPA-soluble and RIPA-insoluble fractions were determined using the BCA assay (Thermo Scientific). The samples were then mixed with sample buffer (25 mM Tris-HCl, pH 6.8, 10% (*v*/*v*) glycerol, 1% (*w*/*v*) SDS, and 5% (*v*/*v*) β-mercaptoethanol) and prepared for analysis by immunoblotting.

### 4.10. Coculture of Neurons with Astrocytes

Primary cortical neurons were isolated from embryonic day (E) 18 rat embryos using established protocols [48]. Briefly, the cortical tissues were dissected, enzymatically dissociated, and mechanically triturated to obtain a single-cell suspension. The dissociated neurons were then seeded at a density of 100,000 cells per well onto primary astrocytes that had been prepared and maintained for at least 8 DIV in 6-well plates. Neurons were cultured in Neurobasal-A medium supplemented with B27, 2 mM GlutaMax, and 100 U/mL penicillin–streptomycin. The co-cultures were maintained under standard cell culture conditions at 37 °C in a humidified incubator with 95% (*v*/*v*) air and 5% (*v*/*v*) CO_2_. At the indicated time points, cells were fixed with 4% paraformaldehyde and 4% sucrose in PBS for 15 min at room temperature. For biochemical analyses, cells were homogenized and processed for immunoblotting.

### 4.11. Statistical Analysis

All experiments were repeated at least three times, unless otherwise stated. All quantitative data are presented as the mean ± standard deviation (SD). Statistical comparisons between control and experimental groups were conducted using two-tailed, unpaired *t*-tests. A *p*-value of less than 0.05 was considered statistically significant for all analyses.

## Figures and Tables

**Table 1:** Primary antibodies used for immunostaining and immunoblotting.

Antibody (Clone No.)	RRID	Host	Assay Dilution	Supplier
GFAP (SMI21)	AB_509978	Mouse	IB: 1:5000	BioLegend (San Diego, CA, USA)
			IF: 1:500	
GFAP-α	AB_10672298	Mouse	IB: 1:5000	NeuroMab (Davis, CA, USA)
			IF: 1:500	
GFAP	AB_2631098	Rabbit	IB: 1:5000	Cell signaling Technology (Danvers, MA, USA)
			IF: 1:500	
panGFAP	AB_10013482	Rabbit	IB: 1:10,000	Dako (Carpinteria, CA, USA)
			IF: 1:1000	
GFAP (GA5)	AB_721051	Mouse	IB: 1:5000	Sigma-Aldrich
			IF: 1:500	
Vimentin (V9)	AB_609914	Mouse	IB: 1:5000	Sigma-Aldrich
			IF: 1:500	
βIII tubulin	AB_2564645	Rabbit	IB: 1:5000	BioLegend
			IF: 1:500	
Actin	AB_10077656	Mouse	IB: 1:5000	Novus (St. Charles, MI, USA)
Pan-neurofilament	AB_2565383	Mouse	IB: 1:10,000	BioLegend
Neurofilament L	AB_823575	Rabbit	IB: 1:5000	Cell Signaling Technology

IB: immunoblotting; IF: immunofluorescence.

## Data Availability

The data that support the findings of this study are available from the corresponding author upon reasonable request.

## Supplementary Materials

The following supporting information can be downloaded at: https://www.mdpi.com/article/10.3390/ijms27104405/s1.
