# Supplementary material for: Synergistic Effects of Glial Fibrillary Acidic Protein Mutation and Overexpression in the Pathogenesis of Alexander Disease
Source: Int J Mol Sci. 2026 May 15;27(10):4405. doi: 10.3390/ijms27104405 (PMC13207236; doi:10.3390/ijms27104405)
Supplement: Supplementary file 1 [file ijms-27-04405-s001.zip › ijms-4285291-supplementary.pdf]

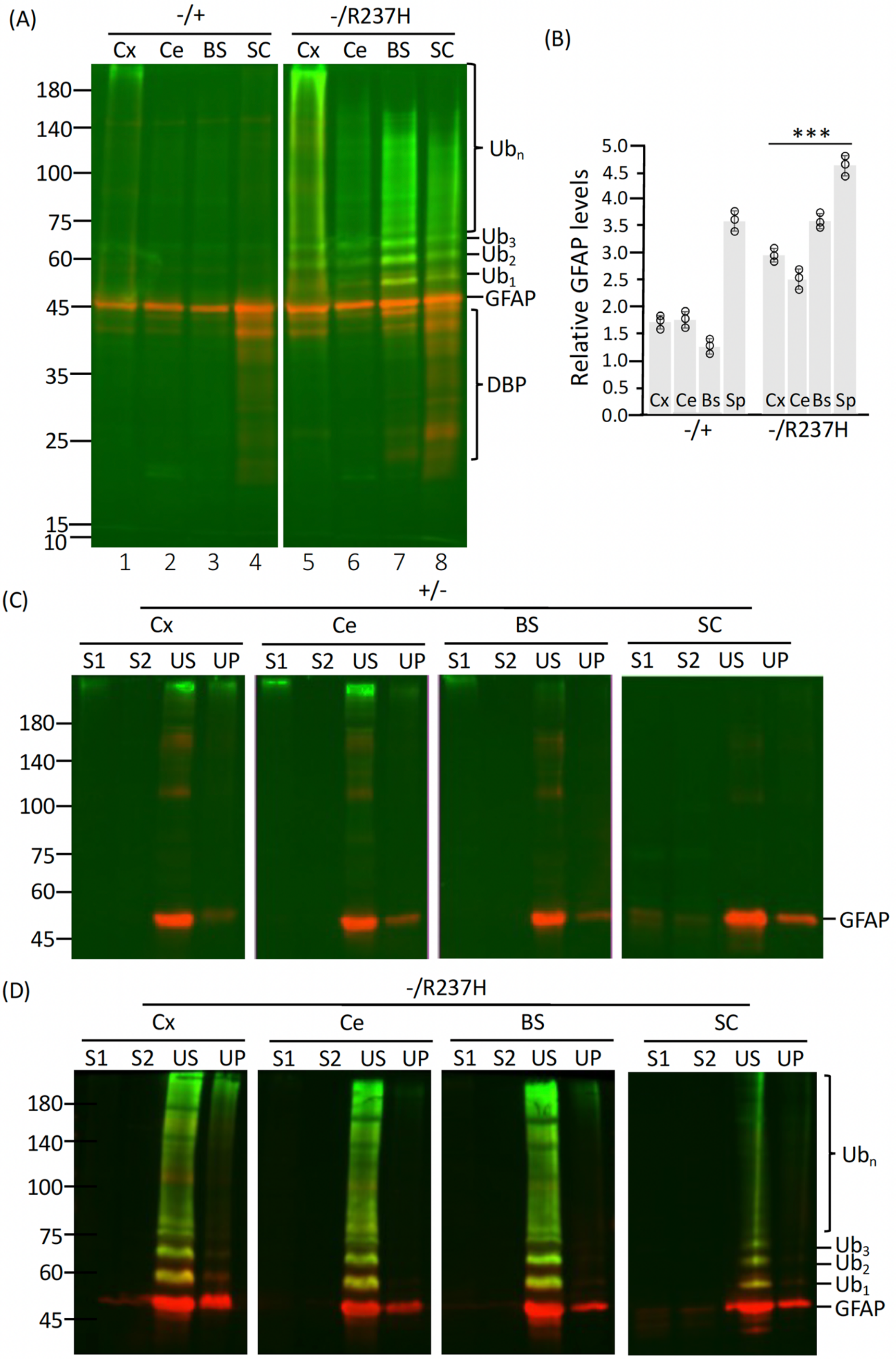

(A)
-/+
-/R237H
Cx Ce BS SC Cx Ce BS SC
180
140
100
75
60
45
35
25
15
10
Ubn
Ub3
Ub2
Ub1
GFAP
DBP
1 2 3 4 5 6 7 8
(B)
***
Relative GFAP levels
5.0
4.5
4.0
3.5
3.0
2.5
2.0
1.5
1.0
0.5
0.0
Cx Ce Bs Sp Cx Ce Bs Sp
-/+
-/R237H
(C)
+/-
Cx Ce BS SC
S1 S2 US UP
GFAP
(D)
-/R237H
Cx Ce BS SC
S1 S2 US UP
Ubn
Ub3
Ub2
Ub1
GFAP

Supplementary Figure S1. GFAP protein levels and solubility properties. (A) Total protein lysates were prepared from CNS regions including cerebral cortex (Cx), cerebellum (Ce), brainstem (BS) and spinal cord (SC) of Het-null (A, lanes 1-4), and Het mutant-null (A, lanes 5-8) rats at 46-58 days of age. Immunoblot analysis was performed using anti-panGFAP and anti-ubiquitin antibodies. (B) Quantification of GFAP levels in tissue lysates of Het mutant-null rats relative to Het-null rats. Each white dot represents a biological replicate (n = 3). Data are presented as mean ± SD. Statistical analysis was performed using a two-tailed t-test; ***$p < 0.001$. CNS tissues prepared from Het-null (C) and Het mutant-null (D) were sequentially extracted into four fractions: S1 (supernatant from detergent extraction), S2 (supernatant from high-salt extraction), US (urea-soluble fraction), and UP (urea-insoluble fraction). Immunoblot analysis was performed on these fractions using anti-GFAP and anti-ubiquitin antibodies. Molecular mass markers (in kDa) are shown on the left, with GFAP and ubiquitinated GFAP species indicated on the right. Ub_{1-3} represent mono-, di-, and tri-ubiquitinated GFAP species, while Ub_n indicates polyubiquitinated GFAP. DBP, degradation breakdown product.

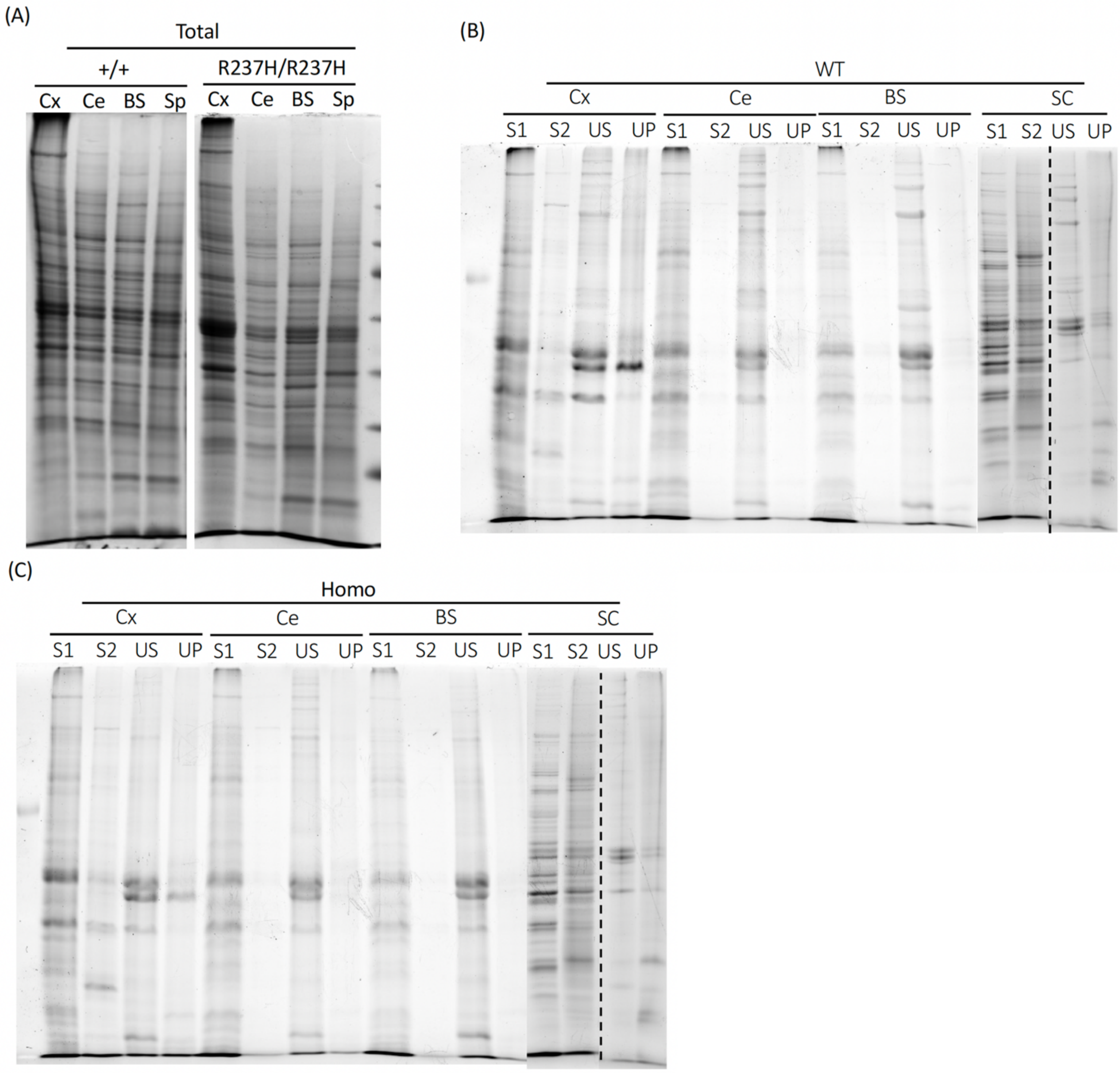

Supplementary Figure S2. GFAP protein levels and solubility properties in homozygous AxD rats. (A) Total protein lysates were prepared from CNS regions including cerebral cortex (Cx), cerebellum (Ce), brainstem (BS) and spinal cord (SC) of WT (+/+, lanes 1-4), and Homo (lanes 5-8) rats at 48-48 days of age. CNS regions prepared from WT rats (B) and Homo rats (C) were sequentially extracted into different fractions: S1 (supernatant from detergent extraction), S2 (supernatant from high-salt extraction), US (urea-soluble fraction), and UP (urea-insoluble fraction). Samples were analyzed by SDS-PAGE and the total protein profiles were visualized by in-gel staining.

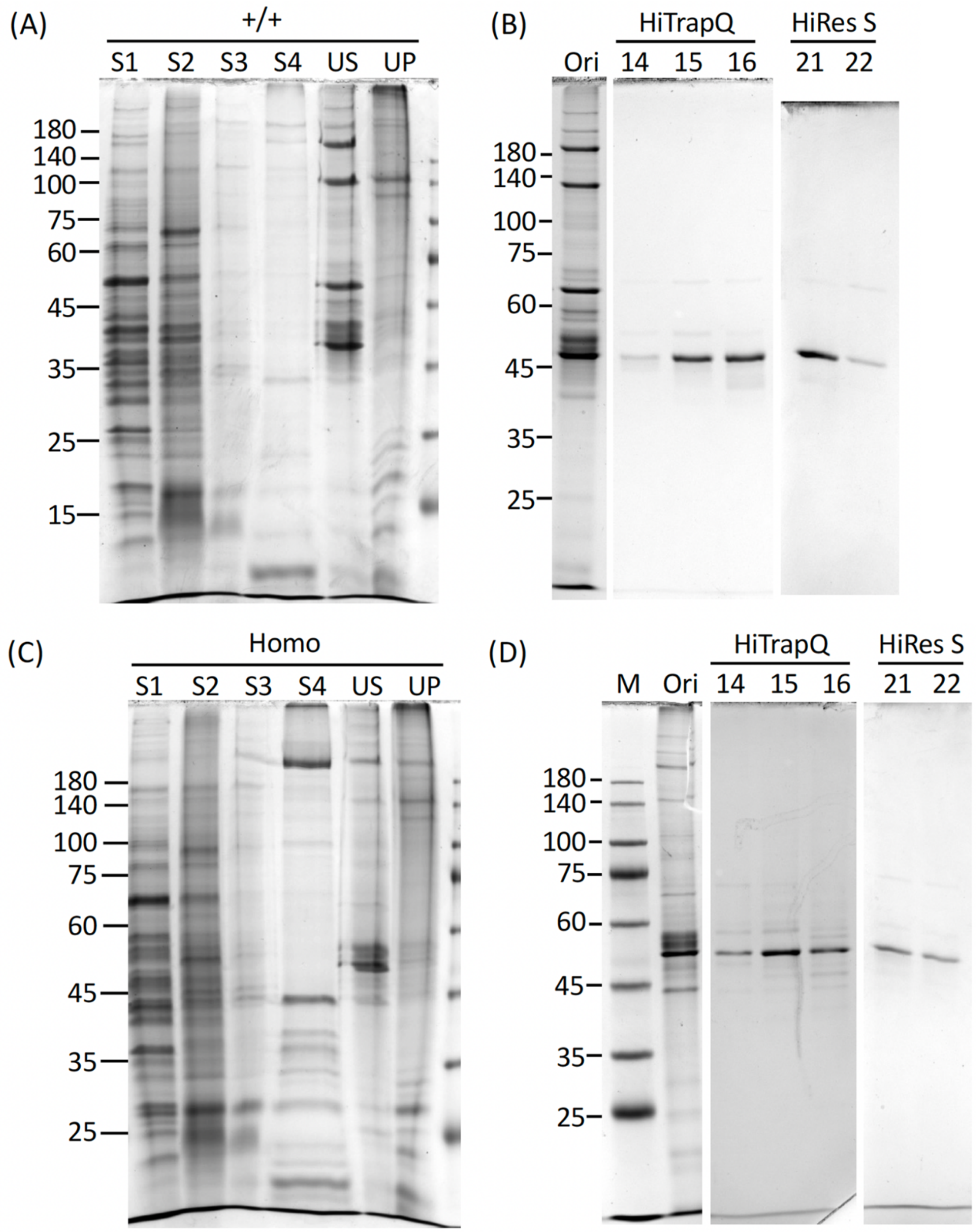

Supplementary Figure S3. Purification of GFAP. The spinal cord prepared from WT (+/+, A) and Homo rats (C) were extracted using an extraction protocol as described in the materials and methods. GFAP enriched in the urea-soluble (labeled US) fraction were further purified by liquid chromatography using HiTrap Q and HiRes S columns (B and D). Selected fractions from each step were analyzed by SDS-PAGE followed by Coomassie blue staining. Molecular weight markers (in kDa) are indicated on the left. M, molecular weight marker; Ori, original sample before purification.

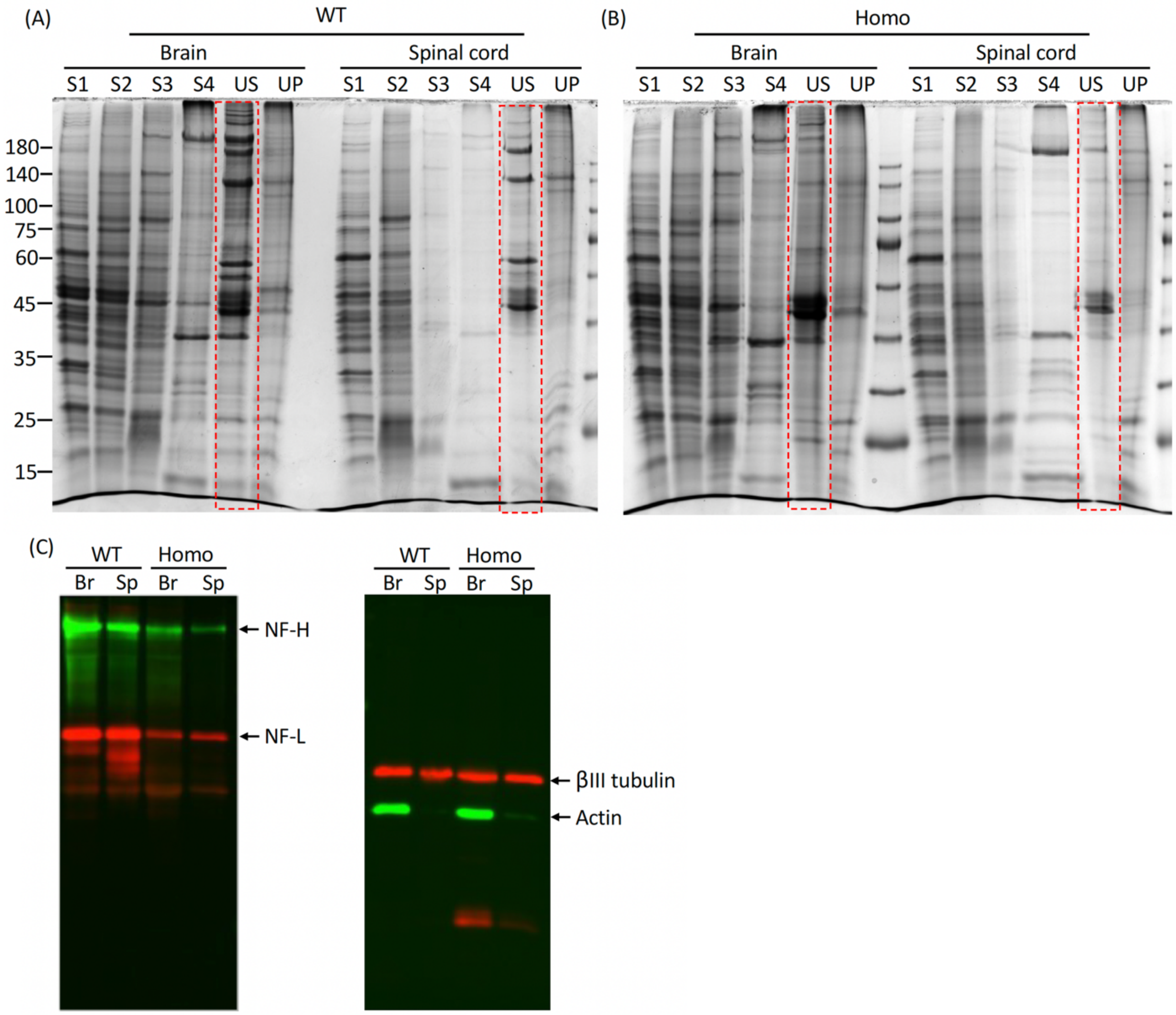

Supplementary Figure S4. GFAP enrichment procedure. Brain (Br) and spinal cord (Sp) prepared from WT (A) and Homo (B) rats were extracted sequentially using buffers with increasing strength. Fractions were analyzed by SDS-PAGE followed by Coomassie blue-staining. Molecular mass makers (in KDa) are shown on the left, and the urea-soluble fractions are indicated by dotted line (labeled red). (C) Uncropped images of immunoblots for Fig. 4 are shown. Samples were analyzed by immunoblotting using antibodies specific to NF-H, NF-L, βIII-tubulin and actin.

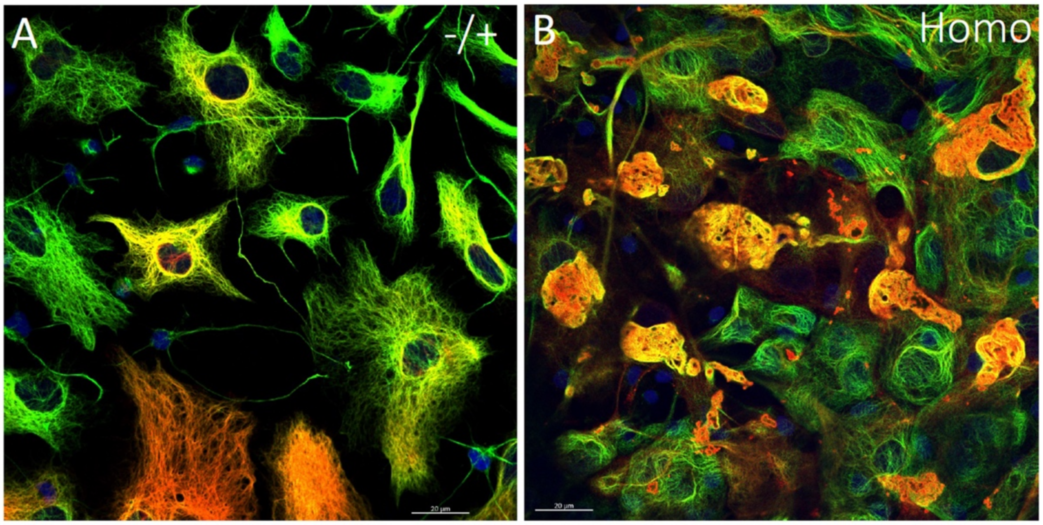

Supplementary Figure S5. Distribution of GFAP in primary astrocytes. (A) Primary astrocytes derived from the Het-null (-/+) rats were cultured for 14 DIV. (B). Primary astrocytes derived from Homo rats were cultured in non-serum medium for 14 DIV. Cells were processed for double label immunofluorescence microscopy using antibodies against GFAP (red channel) and vimentin (green channel). Merged images show colocalization of GFAP and vimentin, with nuclei counterstained using DAPI (blue channel). Scale bar, 20 μm.

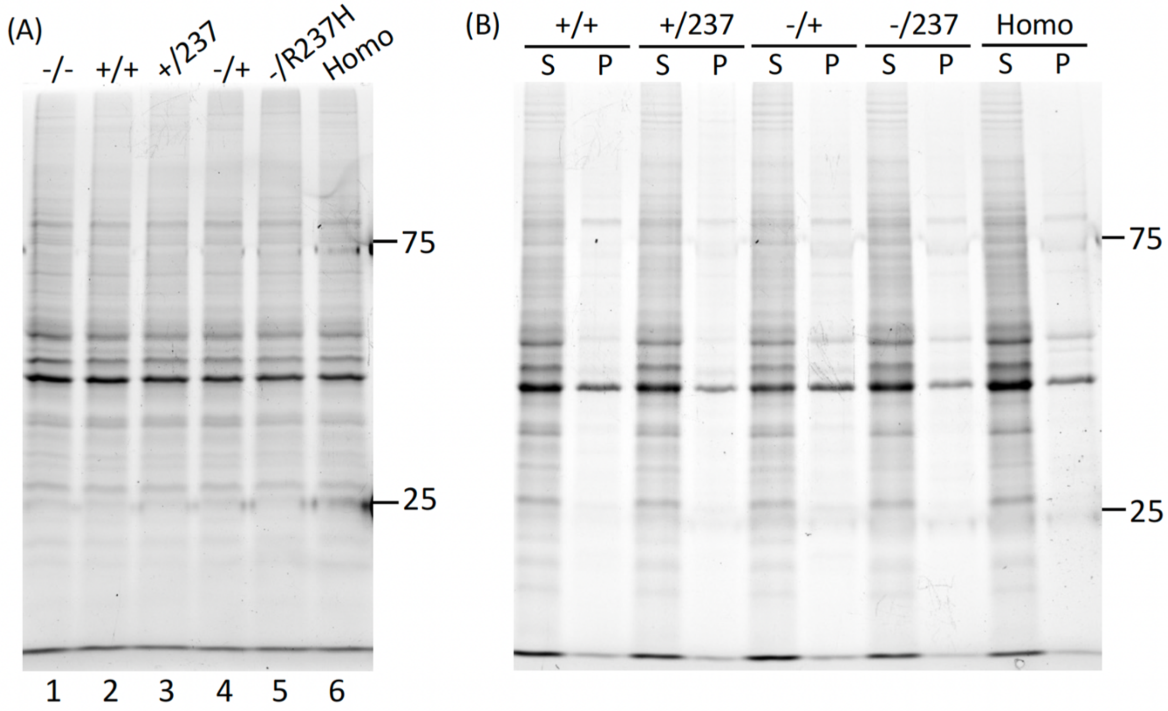

Supplementary Figure S6. Expression levels and solubility properties of GFAP in primary astrocytes. (A) Primary astrocytes derived from rats of the indicated genotypes were extracted with RIPA buffer at 14 DIV. The protein profiles of total cell lysates (A) as well as RIPA-soluble (S) and RIPA-insoluble (P) fractions (B) were analyzed by SDS-PAGE and visualized by in-gel staining. Molecular mass markers (in kDa) are shown on the right.

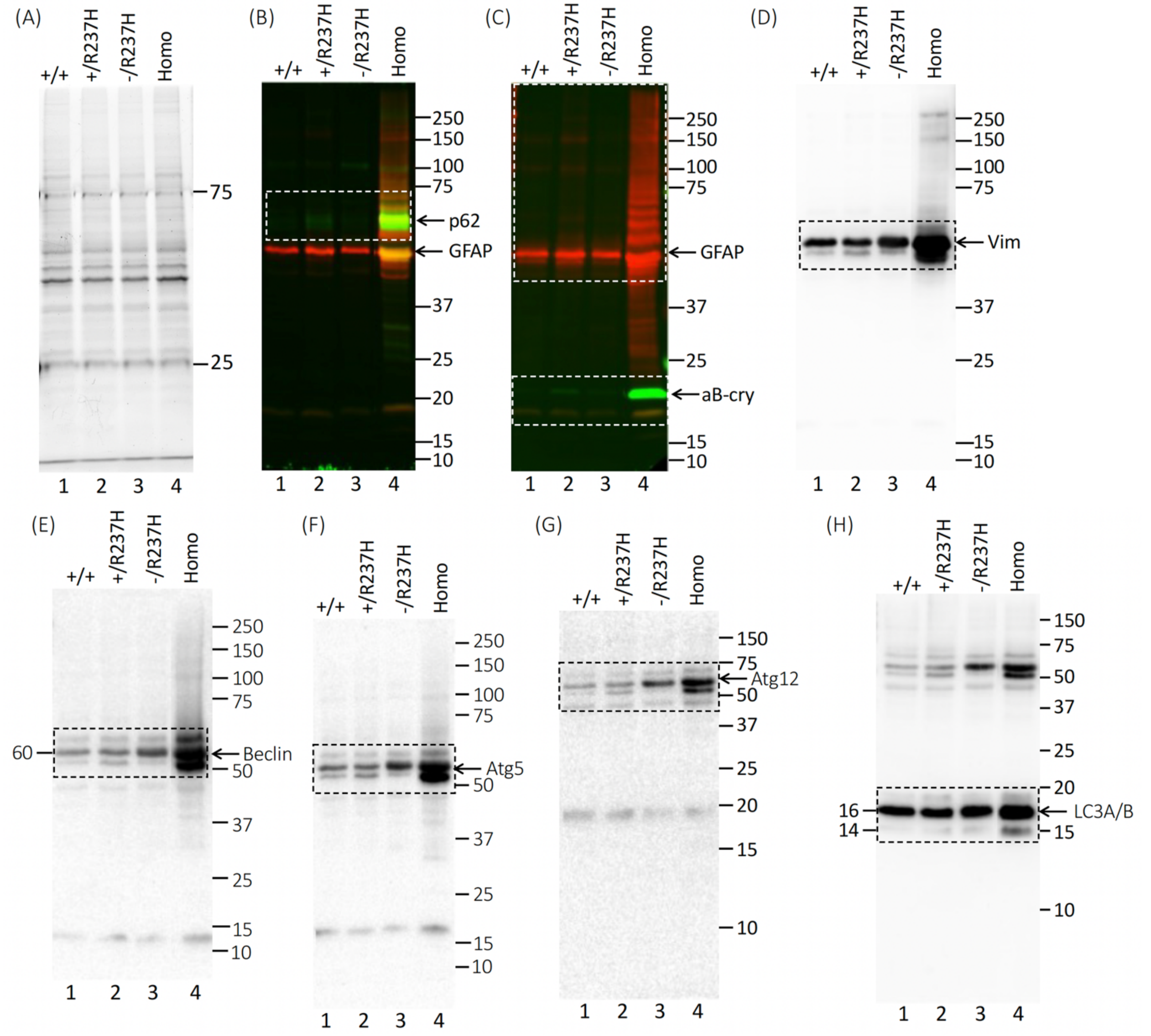

Supplementary Figure S7. Elevated expression of stress and autophagic markers in Homo astrocytes. (A) Primary astrocytes were derived from rats of the indicated genotypes and the RIPA-insoluble fraction were analyzed by SDS-PAGE, followed by in-gel staining. (B-H) Uncropped immunoblot images corresponding to Fig. 7 are shown. Protein samples were analyzed by immunoblotting using antibodies specific to the indicated proteins. Molecular mass markers (in kDa) are shown on the right, along with the positions of the analyzed proteins. Dotted lines indicate regions cropped for data presentation in the main figure.
